# Among the Ancient Hawaiians

**Published:** 1893-10

**Authors:** J. M. Whitney

**Affiliations:** Honolulu, H. I.


### AMONG THE ANCIENT HAWAIIANS.[1]

BY J. M. WHITNEY, D.D.S., HONOLULU, H. I.

To the thoughtful and conscientious dental student and practitioner there must constantly arise questions satisfactory answers to which he may look in vain for in his common surroundings. He

---

[1] Read before the World's Columbian Dental Congress, Chicago, August, 1893.

sees the ravages of dental caries and other diseases so almost universal in our day, and he asks, Is this a necessary evil to which all mankind is subject? or is it a result of the artificial life and varied foods to which our modern civilization binds us? Is dental irregularity due, as some claim, to the mixture of races, and if we could find a people homogeneous and simple, would these conditions exist? Is it true that as mankind advances in the stage of being the third molar is to become gradually eliminated? What is the normal position of adjacent teeth in relation to each other? What relation has the kind of food we use to the building up of dental structure?

We all know the difficulty, amid the complication of modern hygienic conditions, of answering these important questions to our own satisfaction. But if we can examine the dental organs of a simple and isolated people, not affected by the vices and diseases, which are surely not necessarily a contingent of civilization, but which are sure to follow in its train, we may draw some conclusions which will throw much light on our problems. It would be difficult, indeed, for most of us to find living subjects in any number meeting these conditions. But fortunately the bony structures are preserved long after the owners of them have passed beyond mortal ken, and if we can obtain the crania especially of people who, living upon the earth ages ago when wants were few and means of supplying them were correspondingly limited, our study of their dental conditions will certainly be interesting and ought to be instructive.

I consider myself especially fortunate in living in a land where both of these requirements are met; first, the native Hawaiian people, until within a hundred years, lived isolated and unknown to the great world, therefore their habits were simple and their wants and opportunities were few; second, their modes of burial were such that it is possible with comparative ease to obtain some knowledge of their primitive conditions.

"The most isolated place on the globe," that is what Professor Alexander, the learned historian and ethnologist, says of the Hawaiian Islands. Two thousand miles from the Pacific coast of America, and equally distant from the Micronesian, Samoan, and Tahitian groups, the nearest inhabited islands, and more than twice as far from the east coast of China and Australia, they occupy a unique position on the map of the world.

About the year 500 of the Christian era a boat-load of men and women drifted to these shores, either from the Malay Archipelago or from one of the southern islands which had previously been settled by Malays. Except a short period of intercourse between the Pacific Islands in the eleventh and twelfth centuries, these islands were scarcely known to the world until their discovery by Captain Cook, about a hundred years ago. Even the slight intercourse that may have existed during these twelve centuries must have been among kindred races on the Tahitian and Marquèsan Islands, so that practically this people (until within a hundred years) had not changed their race, characteristics, nor their habits of life during the twelve hundred years of their existence. Fortunately, enough of their history and customs have been preserved to give us a good idea of many of their characteristics, their food, etc. They were of medium height, rarely reaching six feet, with heavy strong bones, their crania large and thick. Their employments were tilling the soil, fishing, and warring. Their games were hurling the spear, riding the surf-board, boxing, wrestling, and other exercises requiring great bodily strength and courage.

The climate at the sea-shore averages 75°, with slight variation from day to day, and not varying more than 30° during the year, while as one ascends the lofty mountains with which the country abounds any climate may be found to one of perpetual snow. Thus excessive heat is never found, and vigor of body can be maintained. In the former days of which we have been speaking such diseases as typhus, typhoid, malarial, and scarlet fevers, whooping-cough, measles, mumps, small-pox, syphilis, and leprosy were unknown. Diseases of the alimentary canal and of the lungs were the most prevalent troubles.

Their animal food consisted mainly of fish, with which the sea abounds. Domestic fowl were common, with dogs and swine, both of which were choice articles of food. Of vegetable foods the principal then as now was the *taro* (*Colocasia antiquorum*), which is the Hawaiian "staff of life." From it is made the *poi*, an acid paste, without which a meal is never quite satisfying. They also had yams, sweet-potatoes, and sugar-cane. Their common fruits were cocoa-nuts, bananas, bread-fruit (when cooked resembling sweet-potato), and *ohia*, or mountain apple. Their habits of eating were most irregular, often neglecting to supply themselves with food for several days, and then gorging themselves at any hour of the day or night.

With the incoming of civilization of course many of these conditions have changed. Though *poi* and fish are still the favorite articles of food of the native people, they have added to these many acid and subacid vegetables and fruits, with meat, fine flour, etc.

The second peculiarity of this people rendering a knowledge of their early physical conditions possible is their modes of burying their dead. The most ancient and favorite of these places of interment were in the old lava caves, with which the island of Hawaii particularly abounds. A lava stream flowing from some opening on the mountain-side would cool first on the surface, leaving the still flowing lava within to empty itself on the country below, and thus a long irregular cave of varying dimensions would be formed. Many of these open from mountain-sides, and often from apparently inaccessible precipices. The ancient Hawaiians were very superstitious; the ghost of the dead was supposed to haunt the body long after death, and the friends of the dead anxiously sought the most remote and inaccessible places for depositing their bodies. The islands were teeming with people, and some of these caves are piled many feet deep with the bones of the ancient dead. No wind nor moisture ever reaches them, and the bones are as perfectly preserved as in our most carefully-kept cabinets, after probably hundreds of years since their interment. But the natives even now guard the burying-places of their ancestors with most jealous care, and it is not easy even to one familiar with them and their language to obtain access to these ancient sepulchres.

The other mode of burial to which I referred, and which I consider to be much more recent than that of the caves, was in the sand of the sea-shore. Until within a comparatively few years specimens of crania and other bones from these burying-grounds could be obtained readily in many places. Six or eight miles from Honolulu there was such a place twenty years ago, where for several miles on the sea-shore these human remains lay bleaching in hundreds under the tropical sun, until they had attained the color and texture of ivory. I have seen several similar places on the island of *Oahu* and on *Kauai* particularly, but they have ceased to exist. In some places the grass has grown completely over the sand-mounds, and cattle pasture over whatever bones may remain buried beneath. In others the native people, jealous of their removal, have taken care to break and demolish the skulls, thus rendering them useless. And the South-Sea-Island laborers have sought everywhere for the skulls and removed the teeth for the purpose of making necklaces of them, of which they are very fond.

Realizing more the value of these ancient remains now that they are so difficult to obtain, I recently spent a week on a journey to the island of Hawaii for the purpose of visiting some of these lava caves and securing, if possible, some of their treasures. I was fortunate in being able to obtain the assistance of a friend whose knowledge of the native people and their language made him especially valuable, and I may say indispensable to my success. A voyage of perhaps two hundred miles from Honolulu brought us to the vicinity of the caves. Providing ourselves with candles, stout cord, etc., we at once engaged the service of an old native who claimed to know all about the object of our search, and spent the first day in a vain endeavor to find the ancient cave. Whether the native knew less than he had professed, or whether at the last his courage failed, and he feared the result of guiding us aright, we did not know, but we suspected the latter reason was the true one.

The next day we secured two guides, and after riding many miles over rough lava-covered land we reached a spot which our guides pointed to us as the entrance to a burial-cave. It was near the sea-shore, far from any human dwelling, and from any place that could support a habitation. We could not believe at first that an opening could exist there large enough to admit a man's body, but with much labor we succeeded in removing the rocks so that by considerable effort we were able to force ourselves through. Leaving our unwilling guides at the entrance, we fastened a cord securely to the opening of the cave, lighted our candles, and proceeded to work our way down. Descending among the rocks until we were at least fifty feet below the surface, we suddenly entered a large room, perhaps forty feet high. There were no bodies here, but opening from this room on several sides there were low, narrow passages. Entering one of these, we followed it for, perhaps, a quarter of a mile. Part of the way we were obliged to crawl with great difficulty through the narrow tunnel. We were finally rewarded by again suddenly finding ourselves in a large room, and surrounded on every side by the objects of our search. Near the entrance some of the bodies lay as if hastily deposited, but most of them were laid away with care, some upon shelves partly made with sticks laid in the rock at the side of the cave, more in an opening at the side, which the remains of a stone wall showed to have been at some time walled off from the rest of the cave, while the dim light of our candles showed us several openings in different directions which doubtless led to other similar burial-caves.

The knees were usually drawn up to the breast, tied with a cord, and the whole wrapped in many folds of the native cloth or tapa. By the side of each had apparently been left some food, and perhaps his fish-hook or spear, that he might not want for food in his future home. The air being very dry, and perhaps having some antiseptic property, many of the bodies were completely mummified. Deep dust lay upon everything, and the stillness of death was over all. We could easily imagine with what awe the friends of those lying here had crept down at night and laid away their dead. For the greatest secrecy must be observed, so that no one could ever find them. "I do not wish," said a dying chief, "that my bones should be made into arrows to shoot mice with, or into fish-hooks."

We secured as many specimens of crania only as we were able to carry, packing them in bags. It was growing dark when we emerged from the cave; and when our natives who were waiting outside saw our bags of bones and realized that they must help us to carry them home, they were in utter consternation. It was with difficulty that they could be persuaded to place them upon their horses, and then, regardless of us, of road or path, they took the shortest way home as fast as their horses could carry them, not daring to look behind, lest they should see the pursuing ghosts of their ancestors, leaving us to pick our way as best we could over the rocks in the dark, without even a path, the eight or ten miles to our lodging-place.

We have been taught that primitive peoples, living in simple conditions, were in a great measure free from dental caries as we see it in the mouths of our patients, and that many of the forms of dental disease with which we have to contend were with them wholly unknown. This seems to me a mistaken teaching, as far as may be learned from these records. An exceptional opportunity of becoming acquainted with the crania of the ancient people of these islands during the twenty-four years of my residence here has convinced me that both in the case of those buried in the caves and of those more recent in the sand, not more than twenty-five per cent. have been free from caries, irregularity, or disease. Indeed, I think I have discovered every form of dental disease known to our practice; dental caries in all its many types, necrosis of the teeth, erosion, alveolar abscess, pyorrhœa alveolaris, disease of the antrum of Highmore, necrosis of the maxillary, ankylosis of the jaw, salivary calculus, etc.

Here was a well-developed osseous system; the individual was trained to exercise of the kind that would develop every part of the structure. Living upon an abundance of the simplest yet the most nutritious and bone-developing foods that would not cling to the teeth, but would exercise and clean them, with not an element lacking required by our present knowledge, and yet the same dental disease which we suffer burdened the life of the ancient Hawaiians.

While this is true, I have been interested to find that the teeth of those who died before civilization had introduced to the people peculiar constitutional diseases, acid fruits and vegetables, fine flour, and varied foods, were much less seriously attacked by disease than afterwards. As a general statement the teeth would be found clean, and when caries existed it was here and there in teeth of both maxillaries and on both sides, but not so pervading as found in the more recent crania, or in the mouths especially of the young of the present time.

We have often accounted for the irregularity of teeth found so commonly among Americans by the mixture of races of which our nation is composed. We say that the wide teeth of the large jaw of one race, being crowded into the narrow jaw of another race with which it has mingled, would of necessity produce an irregular arch. But here is a people, isolated from all others for at least fourteen hundred years, with no admixture of races; yet irregularity of the teeth of both maxillaries was almost as common as it is among the mixed races of to-day. It would be difficult to give a good reason why a fixed type for the mouth of this people should not have existed a thousand years ago, and that all with rare exceptions should have been modelled from it, had Nature designed that there should be absolute uniformity in her work.

Among the crania I have examined I have noticed what seemed to be somewhat fixed as a type, that the teeth are set closely together and well rounded, and that the dense part of the enamel, near the cutting-edge or grinding surface, strikes its fellow at that point, the whole being held firmly together by the buttressed third molar.

Perhaps next to dental caries, the greatest source of oral disorders among these people was the irregularity of the third molar, often producing in them as serious consequences as with us of the present time; while its failure to erupt was nearly or quite as common as we find it in our daily practice. So that we cannot argue from these remains, at least, that the coming man is to be deprived of this useful organ.

The relation of food and disease to the health of the dental organs is strongly brought out as we study the changes shown in the teeth of those buried in the oldest caves, and so down through the more recent burials in the sand; then of those who were the old people a quarter of a century ago, whose childhood was passed before civilization had touched their life-habits; and their grandchildren who are now in our schools. These children, as shown by actual examination, have but little better teeth than their white school-fellows. Their fathers and mothers may have better teeth than the children, but it would be an exception if they had not been to the government physician and had one or more teeth removed for relief from odontalgia, while the grandparents, the old men and women whom I found when I first went to the islands, had teeth approximating those found in the old caves, though not as good.

I lay much of this very great change to the many forms of disease that have weakened their constitution, to fine flour that has become a part of their diet, and, eaten in the form of crackers or hard bread, clings to the teeth; to the many acid fruits, such as tamarinds, guavas, limes, etc., to which they have constant access, and to spending their childhood and youth in the school-room instead of wading and swimming in the warm sea, eating raw the fish and shell-fish which they have caught, chewing sugar-cane, and stripping off with their teeth the fibrous covering of the cocoa-nut.

Dr. Whitney stated, after reading his paper, that he had many specimens of crania in which the third molar was not erupted, and he had found that it was as frequent among the ancient Hawaiians not to erupt that tooth as among his present patients.

